# John D. Westbrook Jr (1957–2021)

**DOI:** 10.1107/S2059798321011402

**Published:** 2021-11-01

**Authors:** Christine Zardecki, Stephen K. Burley

**Affiliations:** a RCSB Protein Data Bank, Institute for Quantitative Biomedicine Rutgers, The State University of New Jersey, 174 Frelinghuysen Road, Piscataway, NJ 08854, USA

**Keywords:** obituaries, John Westbrook

## Abstract

John Westbrook is remembered.

John D. Westbrook Jr (1957–2021), Research Professor at Rutgers University and Data & Software Architect Lead for the RCSB PDB, passed away on 18 October 2021. ▸

He was incredibly beloved and respected by his colleagues at Rutgers and worldwide, known for his dry wit and endless enthusiasm for thinking about all aspects of data and data management.

John had a long and highly successful career developing ontologies, tools, and infrastructure in data acquisition, validation, standardization, and mining in the structural biology and life science domains. His work established the PDBx/mmCIF data dictionary and format as the foundation of the modern Protein Data Bank (PDB) archive (wwPDB.org).

More than twenty-five years ago, while still a graduate student, John recognized the importance of a well defined data model to ensure high-quality and reliable structural information to data users. He was the principal architect of the mmCIF data representation for biological macromolecular data. Data are presented in either key-value or tabular form based on a simple, context-free grammar (without column width constraints). All relationships between common data items (*e.g.* atom and residue identifiers) are explicitly documented within the PDBx Exchange Dictionary (https://mmcif.wwpdb.org). The use of the PDBx/mmCIF format enables software applications to evaluate and validate referential integrity within any PDB entry. A key strength of the mmCIF technology is the extensibility afforded by its rich collection of software-accessible metadata.

The current PDBx/mmCIF dictionary contains more than 6200 definitions relating to experiments involved in macromolecular structure determination and descriptions of the structures themselves. The first implementation of this schema was used for the Nucleic Acid Database, a data resource of nucleic acid-containing X-ray crystallographic structures. Today, this dictionary underpins all data management of the PDB. Since 2014, it has served as the Master Format for the PDB archive. It also forms the basis of the Chemical Component Dictionary (https://wwpdb.org/data/ccd), which maintains and distributes small molecule chemical reference data in the PDB.

In 2011, the Worldwide Protein Data Bank (wwPDB) PDBx/mmCIF Working Group was established to enable the direct use of PDBx/mmCIF format files within major macromolecular crystallography software tools to provide recommendations on format extensions required for deposition of larger macromolecule structures to the PDB. This was a key step in the evolution of the PDB archive, which enabled studies of macromolecular machines, such as the ribosome, as single PDB structures (instead of split entries with atomic coordinates distributed among different entry files). In 2019, mandatory submission of PDBx/mmCIF format files for deposition was announced (Adams *et al.*, 2019 ▸).

To ensure the success of the PDBx/mmCIF dictionary and format, John worked with a wide range of community experts to extend the framework to encompass descriptions of macromolecular X-ray crystallographic experiments, 3D cryo-electron microscopy experiments, NMR spectroscopy experiments, protein and nucleic acid structural features, diffraction image data, and protein production and crystallization protocols. These efforts have recently focused on developing compatible data representations for X-ray free electron (XFEL) methods and integrative or hybrid methods (I/HM). I/HM structures, currently stored in the prototype PDB-Dev archive (https://pdb-dev.wwpdb.org), presented new challenges for data exchange among rapidly evolving and heterogeneous experimental repositories. Proper management of I/HM structures in PDB-Dev also required extension of the PDBx/mmCIF data dictionary to include coarse-grained or multiscale models, which will be essential for studying macromolecular structures *in situ* using cryo-electron tomography and other bioimaging methods.

John contributed broadly to community data standards enabling interoperation and data integration within the biology and structural biology domains. His efforts have included (i) describing the increasing molecular complexity of macromolecular structure data, (ii) representing new experimental methodologies, including I/HM techniques, and (iii) expanding the biological context required to facilitate broader integration with a spectrum of biomedical resources. John’s work has been central to connecting crystallographic and related structural data for biological macromolecules to key resources across scientific disciplines. His efforts have been described in more than 120 peer-reviewed publications, one of which has been cited more than 21 000 times according to the Web of Science (Berman *et al.*, 2000 ▸). Eight of his most influential published papers have appeared in the *Inter­national Tables for Crystallography*.

John has also done yeoman service to the crystallographic community over many years and was recognized with the inaugural Biocuration Career Award from the International Society for Biocuration in 2016.

For the International Union of Crystallography, John served on the Commission for Maintenance of CIF Standard (COMCIFS), the Working Group on Data Diffraction Deposition (DDDWG), and the Committee on Data (CommDat). He also served as a Co-editor for *Acta Crystallo­graphica Section F*.

John was a long-standing member of the American Crystallographic Association and served on the Data, Standards & Computing Committee. He also served on the Metadata Interest Group for the Research Data Alliance.

John is survived by his wife, Bonnie J. Wagner-Westbrook, Ed.D. and his devoted Mother-in-Law, Joan N. Wagner of Clinton Twp., NJ; many cousins including Chandler Turner (of Portsmouth, VA), Ann (Turner) Heyes (of Tasmania, Australia), and Louise (Turner) Brown (of Oakland, CA).

## Figures and Tables

**Figure 1:**
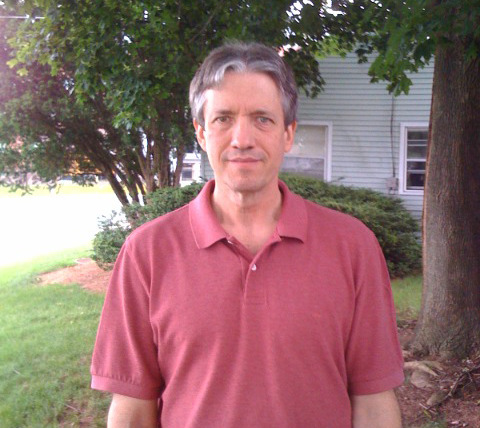
John D. Westbrook Jr.
